# Biomechanical insights into the role of foot pads during locomotion in camelid species

**DOI:** 10.1038/s41598-020-60795-9

**Published:** 2020-03-02

**Authors:** Christofer J. Clemente, Taylor J. M. Dick, Christopher L. Glen, Olga Panagiotopoulou

**Affiliations:** 1 School of Science and Engineering, University of the Sunshine Coast, Sippy Downs, Australia; 2 School of Biomedical Sciences, University of Queensland, St Lucia, Australia; 3 Monash Biomedicine Discovery Institute, Department of Anatomy and Developmental Biology, Monash University, Melbourne, Australia

**Keywords:** Animal physiology, Biomechanics

## Abstract

From the camel’s toes to the horse’s hooves, the diversity in foot morphology among mammals is striking. One distinguishing feature is the presence of fat pads, which may play a role in reducing foot pressures, or may be related to habitat specialization. The camelid family provides a useful paradigm to explore this as within this phylogenetically constrained group we see prominent (camels) and greatly reduced (alpacas) fat pads. We found similar scaling of vertical ground reaction force with body mass, but camels had larger foot contact areas, which increased with velocity, unlike alpacas, meaning camels had relatively lower foot pressures. Further, variation between specific regions under the foot was greater in alpacas than camels. Together, these results provide strong evidence for the role of fat pads in reducing relative peak locomotor foot pressures, suggesting that the fat pad role in habitat specialization remains difficult to disentangle.

## Introduction

All mammalian feet possess a similar role—to transmit the forces associated with locomotion to the environment. However, despite this similar mechanical function, their size, structure, and composition show remarkable variation^1–8^. In some terrestrial mammals, the feet have evolved fatty/fibrous pads or digital cushions that cover bony prominences within the fore and hind feet^7^. However, the primary functions of these pads remain largely unclear. Previous studies have proposed that foot fat pads play various, yet not mutually exclusive roles, such as reducing peak or transient musculoskeletal stresses along the limb^9–12^, reducing localised pressures under the foot^13–17^, or their presence may be related to substrate differences among species habitats^18–20^.

In some terrestrial mammals, including humans, the strain-dependent and compliant properties of fat pads have been shown to play an important role in dissipating and regulating the peak transient loads transmitted to the lower limb during locomotion^7,8,21–27^. This role of fat pads in dissipating and distributing the mechanical forces associated with locomotion likely becomes even more pivotal as animals increase in size or move faster. Size becomes important because scaling patterns predict that stress and pressure become disproportionally large as animals increase in body mass^2,10–12,28–31^. Stress and pressure are equal to force divided by area; thus locomotor pressures and stresses could be moderated by reducing the forces applied, or by increasing the surface area over which the forces act. Geometric scaling predicts that locomotor forces increase proportionally with body mass (M^1.0^), yet the cross-sectional area available to withstand these forces increases with a lower exponent (M^0.67^), predicting stress to increase as M^0.33^. Fat pads may decrease this stress/pressure by reducing the loading rate and therefore the peak impact forces. For example, in humans, fat pads located under the heel have been shown to attenuate 50–90% of the heel strike impact by the time the stress wave reaches the knee, and as much as 98% when the wave reaches the head^22,32–34^. Moreover, humans often rely on artificial fat pads in the form of running shoes to aid in the minimization of musculoskeletal stresses and foot pressures associated with high speed locomotion^33,35^.

An alternative solution to reduce stress and pressure is to increase the area over which locomotor forces act. Fat pads may distribute and thus reduce high, localised pressures (and loads) which may otherwise cause damage to the foot if their magnitudes exceed tissue safety thresholds. For example, the world’s heaviest extant terrestrial mammal, the African elephant has prominent fat pads located posterior to their toes (centrally and caudally to the feet). We have previously shown that the size of these pads increases with body mass to allow localised peak pressures around the fat pad to remain constant throughout ontogeny^14^. In captive elephants and rhinoceroses, regions of peak pressures correlate with skeletal pathologies suggesting that adaptations, such as enlarged fat pads, may be a mechanism to minimize localised regions of high pressure that could cause tissue damage^14–16^. However, it remains unknown the extent to which the presence of these fat pads allows large-bodied mammals to deal with both size- and speed-associated increases in musculoskeletal force and foot pressures.

The presence of fat pads may also be related to differences in the type of substrate on which a species typically moves. For example, the broad leathery fat pads in camels have been suggested to function to disperse their weight on a wider surface area to avoid their feet sinking into loose sandy soil^36^ or even snow^37^, however to our knowledge, neither is yet to be experimentally confirmed. In dune dwelling geckoes, variation in foot morphology suggests that webs and fringes on toes act to increase surface area to aid in locomotion on sand, yet both also appear to facilitate movement in the substrate or in burrowing^38^. Further, other large desert adapted mammals such as the Oryx appear to lack large fat pads^39^. Therefore, it can be difficult to disentangle the relative roles of fat pads in foot pressure mitigation or habitat specialization, especially since they have previously been studied in such diverse groups.

**Figure 1:**
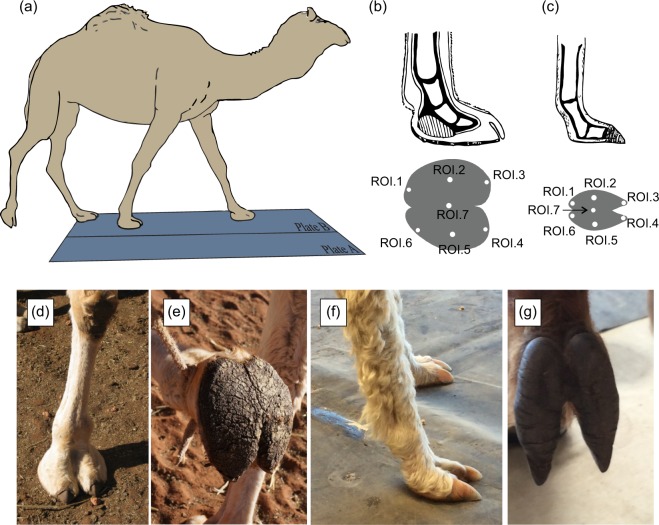
(**a**) Schematic illustration showing the position, and relative size, of the pressure plates during experimental data collection. The dimensions of each plate were 0.605 m (width) and 2.122 m (length) with a 48 (width) by 160 (length) array of sensors to total 7680 sensors for each plate. Location of the 7 regions of interest (ROI) in (**b**) camel and (**c**) alpaca foot. Position of the fat pad in the camel foot is indicated by the vertically shaded structure. Photographic illustration of the alpaca and camel feet. (**d**) Sagittal view of the camel fore right foot. (**e**) Palmar view of the camel fore right foot. (**f**) Frontal view of the alpaca fore left foot. (**g**) Palmar view of the alpaca fore left foot.

Camelids are the ideal family to study the function of fat pads because within this group we have examples of well-developed and under-developed fat pads among closely related species that share the same unique camelid didactyl foot skeleton (Fig. 1). Within camelids, the largest species, the dromedary camel (*Camelus dromedarius*, mass = 400–600 kg) has feet which possess two broad, oval-shaped pads separated by an interdigital septum (Fig. 1). Thus when walking, the portion of the foot that contacts the substrate consists of two digits surrounded by the large cushioning pad (Fig. 1)^40–42^. Yet the second smallest species of camelids, the Suri alpacas (*Vicugna paccos*, mass = 55–65 kg) have a comparatively reduced fat pad, consisting of a small single fat pad on each digit, connected solely by an interdigital link near the proximal interphalangeal joint^43^. These species also vary in habitat, with camels typically inhabiting soft, unstable sandy substrates found in the open, arid environments^44,45^. Alpacas, however, inhabit a broad range of environments, including harsh arid mountain regions, savannah and temperate forest^45^, thus their narrower footpads may reflect a more generalist functional morphology adapted for the broader range of their habitats. These specific morphological differences between related species with limbs that are otherwise structurally similar, allow us to determine how the presence of fat pads may relate to mitigating size- and speed-related pressures, or habitat variation, without the confounding effects of large phylogenetic differences.

The aim of this study is to determine within and between species scaling of vertical ground reaction force, foot contact area, and foot pressure during locomotion in camelids. We examined 701 footfalls from eight camels and 1954 footfalls from twelve alpacas while moving over customized pressure platforms to provide a detailed examination of vertical ground reaction forces, foot contact areas, and time-varying pressures. If we assume similar kinematics between camels and alpacas, we can make several hypotheses regarding the role of fat pads. If fat pads play a prominent role in reducing peak foot pressures, we would expect camels (possessing comparatively larger pads) to show lower mass-specific peak pressures at similar speeds, in comparison to alpacas. Additionally, if fat pads play a prominent role in reducing localised pressures, we would expect less variation in peak pressures at analogous regions underneath the camel foot, in comparison to the alpaca foot. Alternatively, if fat pads do not appear to cause a significant reduction in locomotor pressures, it may provide evidence for a habitat-specific modification linked to locomotion over loose granular media, such as sand or snow.

## Results

The walking speeds of alpacas and camels varied from 0.44 m s^−1^ to 1.85 m s^−1^ and 0.98 m s^−1^ to 2.466 m s^−1^, respectively. This corresponds to Froude numbers^46^ (Fr = velocity^2^ × [9.81 m s^−2^ × hip height]^−1^) ranging from 0.0043 to 0.892 (avg. = 0.1757; n = 480) and 0.0397 to 0.388 (avg. = 0.129; n = 453) for alpacas and camels, respectively (Supplementary Tables 1 and 2). Visual analysis of the walking sequences from video recordings indicated that both camels (Supplementary Video 1) and alpacas (Supplementary Video 2) predominantly used a same-side diagonal gait.

We examined vertical ground reaction forces, contact areas, and foot pressures for camels and alpacas of varying body masses as they moved at different walking speeds across the pressure plates. We hypothesized that if fat pads play a prominent role in reducing locomotor foot pressures, camels will show lower mass-specific peak pressures and relatively higher foot contact areas as both mass and speed increases, in comparison to alpacas.

**Figure 2:**
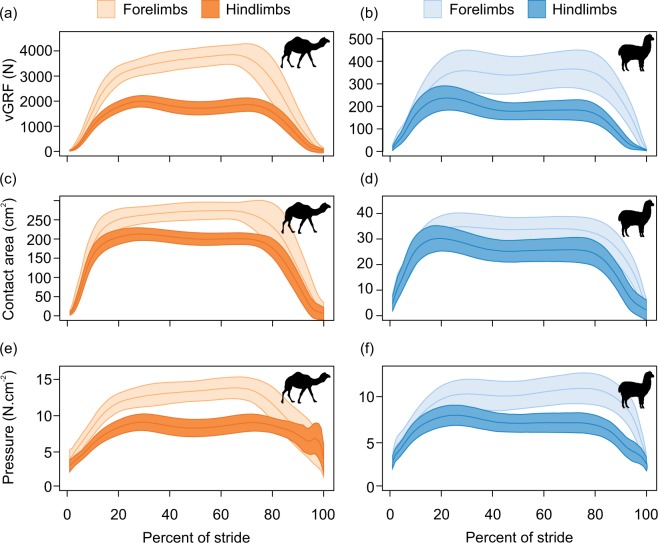
Time-varying vertical ground reaction forces (vGRF; **a**,**b**), foot contact areas (**c**,**d**), and pressures (**e**,**f**) for camels (left) and alpacas (right). Forefeet are shown in lighter colours and hindfeet in darker colours. Lines represent means ± SE.

A comparison of loading regimes in the fore- versus hindlimbs revealed similar patterns between camels and alpacas. Within camels there was significant variation in peak vertical ground reaction force between the fore and hindlimbs (F_1,619_ = 15,827, *p* < 0.001) but not between the left and right fore or hind feet (Fig. 2a). This suggests that at slow locomotor speeds, camels load their forelimbs (3949 ± 116 N) more than their hindlimbs (2016 ± 75 N), indicating a 1.9 times greater peak load on the forelimbs in comparison to the hindlimbs. Similarly, in alpacas, there was significant variation in peak vertical ground reaction force between the fore and hindlimbs (F_1,1940_ = 9266, *p* < 0.001) but not between left and right fore or hind feet (Fig. 2b). This suggests that at slow locomotor speeds, alpacas load their forelimbs (389 ± 25 N) more than their hindlimbs (245 ± 14 N), indicating a 1.6 times greater peak load on the forelimbs.

Patterns of force, area and pressure throughout the stance are presented in Fig. 2. Both species show a pattern consistent with an initial foot strike peak, followed by a period of flattened or reduced, force/area/pressure, followed by a final push off peak. The pattern for alpacas appears more pronounced with the impact peak tending to be higher than the push off peak, though this was not the case for camels.

Given the higher peak forces in forelimbs in both species, the remainder of the analysis focuses on variation in forelimb mechanics. Analysis for the hindlimbs is presented in the supplementary materials section but the trends identified for the hindlimbs closely match those outlined below for the forelimbs.

### Vertical ground reaction forces during movement

**Figure 3:**
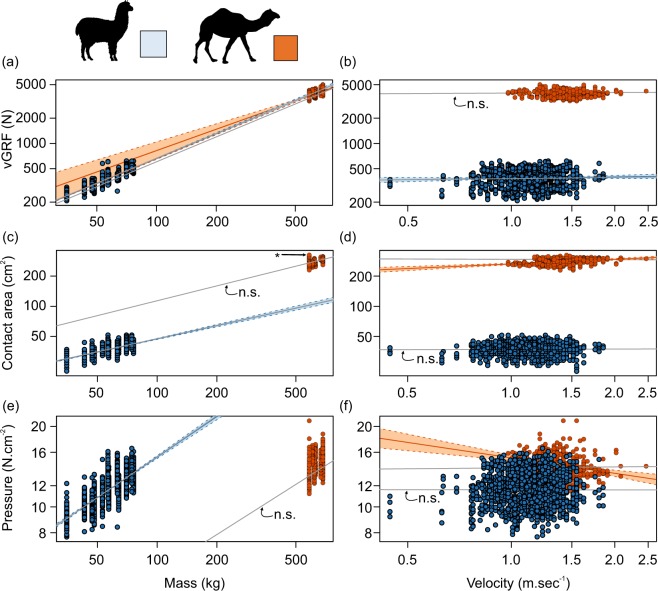
Relationship between mass (left) and velocity (right) with (**a**,**b**) vertical ground reaction force (vGRF), (**c**,**d**) contact area, and (**e**,**f**) relative pressure for camels (orange) and alpacas (blue). The thinner solid coloured lines indicate linear regression models for each group with 95% confidence intervals shown as the shaded regions bounded by the dashed lines. The thicker solid grey lines indicate the result of the linear mixed-effects models, including subject as a random factor.

When including mass, velocity, and species in a linear mixed effects model, forelimb peak vertical ground reaction force was significantly affected by mass (F_1,16_ = 11,946, *p* < 0.001) and velocity (F_1,1684_ = 16.0, *p* = 0.001), but not species (F_1,16_ = 0.1, *p* = 0.729). Further, there was a significant interaction between velocity and species (F_1,1684_ = 7.6, *p* = 0.006) but no other interaction was significant. To explore this further, we looked at the effects of mass and velocity on forelimb peak vertical ground reaction force in each species independently. Among camels, peak vertical ground reaction force increased with M^0.98^ (CIs: 0.31–1.66) whilst in alpacas, peak vertical ground reaction force increased with M^0.98^ (CIs: 0.84–1.12) (Fig. 3a). Among camels, there was no effect of velocity on peak vertical ground reaction force (slope velocity: 0.02; CIs: −0.03–0.07) whilst in alpacas, peak vertical ground reaction force increased very slightly, but significantly with increases in velocity (slope velocity: 0.04; CIs: 0.02–0.06) (Fig. 3b). Similarly, when we normalized peak vertical ground reaction force for body mass, there was no significant effect of velocity on mass-corrected peak vertical ground reaction force in camels (velocity slope: 0.02; CIs: −0.04–0.07) while for alpacas we found that mass-corrected vertical peak ground reaction force increased with velocity (velocity slope: 0.05; CIs: 0.03–0.07).

### Foot contact area during movement

Exploring peak contact area while including mass, velocity, and species in a linear-mixed effects model revealed a significant effect of mass (F_1,16_ = 9558, *p* < 0.001) and species (F_1,16_ = 47, *p* < 0.001), but no effect of velocity (F_1,1684_ = 0.68, *p* = 0.409). Further, there was a significant interaction between velocity and species (F_1,1684_ = 13.3, *p* < 0.001) but no other interaction was significant. To explore this interaction further, we analyzed the effects of mass on foot contact area in camels and alpacas separately. Camels and alpacas show a similar increase in foot contact area with body mass (Fig. 3c). Among camels, contact area increases with M^0.49^ (CIs: −0.24–1.22), but this effect was not significant (*p* = 0.15). However, this pattern is largely driven by a single individual who displays markedly large foot contact areas for its small body mass (Fig. 3c, [“*”]). When removing this individual, contact area significantly increases with M^0.90^ (CIs: 0.46–1.33; F_1,5_ = 28, *p* = 0.003). Among alpacas, contact area increased significantly with M^0.44^ (CIs: 0.32–0.55; F_1,10_ = 69, *p* < 0.001). Yet the intercept of the relationship between contact area and mass was greater in camels than alpacas, indicating that at any given body mass, foot contact area is greater in camels than alpacas (Fig. 3c).

Further comparing contact area with velocity among camels using linear mixed effects models, revealed a non-significant increase in contact area with velocity (velocity slope: −0.01, CIs: −0.04–0.02, F_1,389_ = 0.52, *p* = 0.471) however this increase does become significant when using standard linear models (velocity slope: 0.15, CIs: 0.11–0.20), F_1,396_ = 44, *p* < 0.001) (Fig. 3d) suggesting the small sample size for camels may be the cause of large confidence intervals for these linear mixed effects models. In alpacas, linear mixed effects models (velocity slope: 0.01, CIs: −0.02–0.03, F_1,1297_ = 0.50, *p* = 0.480) and standard linear models (velocity slope: 0.02, CIs: −0.02–0.06, F_1,1308_ = 1.19, *p* = 0.274) agree, and both suggest a non-significant effect whereby contact area does not change with velocity (Fig. 3d).

### Pressures during movement

Combining force and contact area data to understand pressure variation with mass, velocity, and species revealed a significant effect of mass (F_1,16_ = 85, *p* < 0.001) and species (F_1,16_ = 32, *p* < 0.001), but no effect of velocity (F_1,1684_ = 0, *p* = 0.967), nor any interaction between these variables. This significant effect of species suggests that camels have a relatively lower pressure at any given mass or velocity, when compared to alpacas (Fig. 3e). The significant effect of mass, but the lack of a significant interaction between mass and species suggests that we cannot significantly differentiate the slope between pressure and mass in camels versus alpacas. Yet when we examine the camels independently from the alpacas, among camels there was no significant relationship between mass and pressure (M^0.47^, CIs: −0.43–1.37, *p* = 0.251), whereas in alpacas, pressure significantly increased with mass (M^0.51^, CIs: 0.35–0.66, F_1,10_ = 54, *p* < 0.001 (Fig. 3e).

Comparing pressure and velocity independently for camels using linear mixed effects models, revealed a non-significant decrease in pressure with velocity (velocity slope: 0.01, CIs: −0.05–0.08, *p* = 0.732) but this reduction in pressure with velocity does become significant using standard linear models (velocity slope: −0.19, CIs: −0.26-(−0.12), F_1,396_ = 30, *p* < 0.001) (Fig. 3f) again suggesting the small sample size for camels may be the cause of large confidence intervals for the linear mixed effects models. As for contact area above, in alpacas, linear mixed effects models (velocity slope: −0.001, CIs: −0.02–0.02, *p* = 0.913) and standard linear models (velocity slope: 0.02, CIs: −0.02–0.06, *p* = 0.277) agree, and both suggest a non-significant effect whereby pressure does not change with velocity (Fig. 3f).

### Localised pressures under the foot during movement

**Figure 4:**
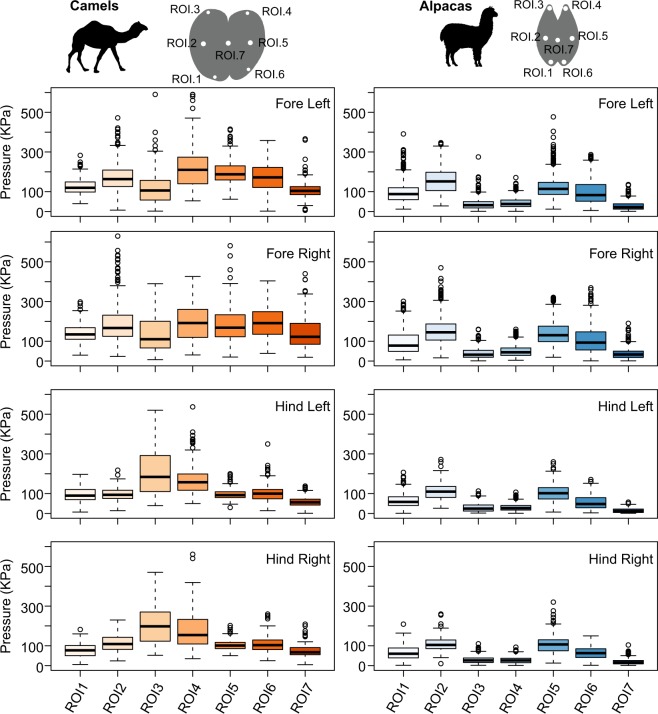
Patterns of peak pressure distributions during the stance phase for the feet of camels (left) and alpacas (right). Camels more evenly distribute forces under their feet during locomotion. In the fore and hind feet, camels display the greatest pressures in the anterior region (ROI3 and ROI4), whereas alpacas display the greatest pressures in the mid-region of their feet (ROI2 and ROI5). Regions of interest (ROIs) are shown for each foot, and the variation among them is shown in the box plots. Data represents the average peak pressure in each foot for all subjects and strides. Boxes represent the median, with hinges representing the first and third quartiles; whiskers represent the 95% CIs, and dots represent outliers.

We examined differences in regional patterns of loading within each of the feet and between alpacas and camels. Camels more evenly distribute forces under their feet during the stance phase of locomotion as indicated by less variation in pressure among ROIs under individual feet (Fig. 4).

In camels peak pressure during stance varied significantly with ROI (F_6,4859_ = 118, *p* < 0.001) and among the feet (F_3,4859_ = 150, *p* < 0.001), with a significant interaction between the terms (F_18,4859_ = 37, *p* < 0.001) (Fig. 4, left panel). The lateral anterior portion (ROI4) in the fore feet showed moderately higher peak pressures (208.6 ± 7.3 KPa) compared to other regions of the foot, however pressures were similar across all regions. In the camel hind feet, the highest peak pressures were in the lateral and medial anterior portions (ROI3 and ROI4) (205.5 ± .8.4 KPa and 177.8 ± .7.5 KPa) with all other ROIs showing lower pressures.

Similarly, in alpacas peak pressure during the stance phase varied significantly with ROI (F_6,13259_ = 1859, *p* < 0.001) and among the feet (F_3,13,259_ = 355, *p* < 0.001), with a significant interaction between the terms (F_18,13259_ = 17, *p* < 0.001) indicating that the regional patterns of loading vary within and among the feet (Fig. 4, right panel). Post hoc analyses revealed that in the alpaca fore feet, all ROIs displayed significant variation from one another. In the alpaca hind feet, all pairs of ROIs varied significantly with the exception of ROI1 versus ROI6, ROI2 versus ROI5, and ROI3 versus ROI4. The medial posterior and middle (ROI1 and ROI2) and lateral posterior and middle (ROI5 and ROI6) regions of the forefeet showed moderately higher peak pressures than anterior (ROI3 and ROI4) and central (ROI7) regions. In the fore feet, the medial mid-region of the foot (ROI2) displayed the highest peak pressure (154.1 ± .2.6 KPa). In the hind feet, however, the highest peak pressures were in mid-lateral and medial regions (ROI2 and ROI5) (ROI2 = 110.3 ± .2.1 KPa and ROI5 = 106 ± .2.5 KPa), with the posterior region (ROI1 and ROI6) showing moderate pressures, and the anterior (ROI3 and ROI4) and central (ROI7) aspects of the feet exhibiting the lowest pressures.

A comparison of loading patterns in alpacas versus camels illustrates that camels more evenly distribute pressure across their fore and hind feet (Fig. 4). Post-hoc results indicate that, among forefeet ROIs in alpacas, all 21 pair comparisons were significantly different. In contrast, for camels forefeet only 13 of the 21 comparisons varied significantly among ROIs, suggesting less variation in pressure. The differences were smaller in the hind feet, with 18 of the 21 comparisons significantly different in alpacas while 17 of the 21 were significantly different in camels –again suggesting slightly greater variation in regional loading patterns in alpacas. Additionally, there are distinct differences in the distribution of pressures across the feet in alpacas versus camels. In both the fore and hind feet, alpacas display the greatest pressures in the mid-region of their feet (ROI2 and ROI5) whereas camels display the greatest pressures in the anterior region (ROI3 and ROI4).

## Discussion

The fat pad of the vertebrate foot varies markedly in size and composition, yet the links between the fat pad’s morphology and its biomechanical function remains unclear. This is likely a result of different functional, developmental, and evolutionary constraints on fat pad design—making relationships between form and function often difficult to disentangle. The camelid family provides a useful experimental paradigm to explore the fat pad form-function links, as within this phylogenetically constrained group we see examples of species with enlarged fat pads (camels) and others where fat pads are greatly reduced (alpacas). In this study, we explored the extent to which fat pads may alleviate the foot pressures associated with increases in body mass or movement speed by comparing camels and alpacas.

All else being equal, larger animals are expected to have higher peak stresses during locomotion in comparison to smaller animals –due to the disproportionate scaling of force and cross-sectional area. Similarly, increases in running speed would be expected to increase the forces transmitted to the ground, resulting in higher stresses and foot pressures. Our results show that the effect of body mass on peak vertical ground reaction force, for both camels and alpacas, scaled as expected based on geometric similarity, near M^1.0^, yet differences arise when examining the influence of velocity. For alpacas, we found increases in velocity resulted in a significant increase in vertical ground reaction force, yet for camels, only a weak non-significant increase in vertical ground reaction force was evident.

We found that in both camels and alpacas, foot contact area increased with body mass, but camels displayed a higher intercept, suggesting that at any body mass, camels have relatively broader feet –as has been previously suggested^30^. We also show, to our knowledge for the first time, that foot contact area increased with movement speed for camels but was invariant for alpacas. This highlights that when camels increase movement speed, foot contact area increases, but this is not the case for alpacas, presumably a result of the visco-elastic properties of the fat pad which is exaggerated only in the former species. Fat pads are composed of a collagenous outer layer filled with a viscous gel-like tissue that is threaded with elastic fibres spanning the internal structure. These elastic fibres can stretch during the stance phase of a stride, and then recoil to reset the shape of the cushion after the deformation that occurs^43^. This ability to increase contact area in response to velocity could be a foot pressure reduction mechanism, and our results indeed support this. We show that in camels, foot pressure does not significantly increase with body mass, but in alpacas it does. In fact, with increases in velocity, there was even a weak decrease in foot pressures in camels, but no change in alpacas. Thus, similar to humans wearing running shoes, the fat pads in camels appear to reduce the pressures associated with locomotion.

**Figure 5:**
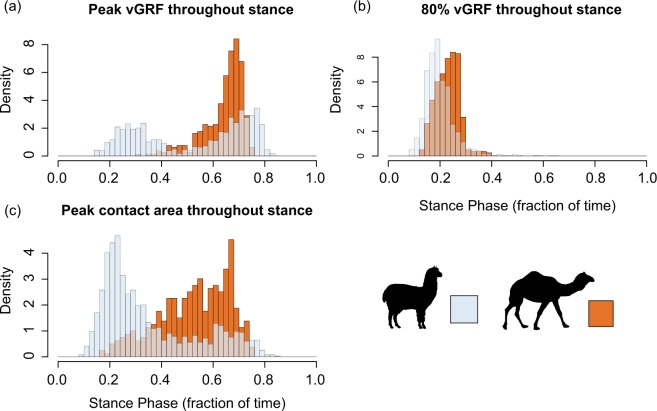
Density histograms (relative frequency), expressed as a fraction of stance phase duration, for (**a**) timing of peak vertical ground reaction force (vGRF) during stance, (**b**) timing to reach 80% of peak vGRF during stance and (**c**) timing to reach peak contact area during stance for camels (orange) and alpacas (blue). (**a**) shows that alpacas have a bi-modal distribution of peak vGRF with both an early and a late peak, whereas camels show only the latter peak. (**b**), the 80% of vGRF allows us to compare the initial loading rate given this bi-modal distribution in alpacas, and late peak in camels.

In humans, running shoes have been shown to reduce not only the total peak stresses, but also the early transient peak forces associated with heel strike^33^. When comparing the biomechanics of camels to alpacas, we see a similar effect as for shod versus barefoot human running, whereby the initial impact peak is present in alpacas, but is reduced in camels. The histogram in Fig. 5 shows the timing of peak vertical ground reaction force over the stance phase, and while alpacas show a bi-modal distribution with both an early and a late peak, camels show only the latter peak, which is likely associated with the push-off of the stance phase. This difference in vertical ground reaction force profiles between camels and alpacas may indicate a modification in loading regimes as a result of the fat pad. To explore this further, we plotted the time to reach 80% of peak vertical ground reaction force, which would indicate how the initial loading rate varies between the species (Fig. 5b). The 80% of peak vertical ground reaction force for alpacas occurs earlier than that for camels, indicating that forces increase more rapidly in the former species when compared with the latter. These variations show a function of the fat pad in reducing not only peak loads during stance, but an effect of reducing the rate of loading, similar to previous results for humans wearing running shoes^35^.

The association between reduced peak vertical ground reaction forces and the presence of enlarged fat pads may suggest a strong form-function link. Fossil evidence from small-bodied early elephants (stem Proboscidea), suggests that the foot bones were held relatively flat and had no evidence of an enlarged fat pad^47^. As larger body masses evolved among elephants and they became more terrestrial, their foot posture may have become more plantar-flexed and evidence suggests they evolved enlarged fat pads along with predigits^47^. Similar trends have been proposed for sauropod and ornithopod dinosaurs, with evidence for correlated evolution of larger fat pads, and more digitigrade foot postures associated with increases in body mass^48,49^.

Yet an alternative, though not mutually exclusive, function of the fat pad may be to distribute forces more evenly underneath the foot to avoid high localised foot pressures at any one region. Localised foot pressures in large bodied animals may result in foot pathologies such as inflammation, enthesopathies, cracks, defects and horn growth that could impact the animals’ health and ability to locomote effectively e.g.^50–52^. Although many factors likely contribute to foot pathologies, exposure to hard surfaces (asphalt, concrete) can further compromise the effectiveness of the fat pad and accelerate these pathologies^53–55^. The exact mechanisms under which hard substrates can disrupt the fat pad is not well understood, but if the fat pad plays a role in reducing localised peak pressures, then we would expect species with reduced fat pads to display higher localised regions of pressure compared to species with well-developed fat pads. This appears to be the case for the comparison between alpacas and camels. We found that among the forefeet ROIs in alpacas, all 21 pair comparisons were significantly different, despite the limitations in the force plate design resulting in resulting in a relatively poorer spatial resolution for this species. This suggests alpacas show large variation in pressures across different regions underneath the foot. In contrast, for camels only 13 of the 21 comparisons among ROIs varied significantly, suggesting less variation in pressure underneath the forefeet in camels, with consistent results for the hindfeet. Similar to our previous studies in elephants and rhinoceroses^14–16^, in camels the peak pressures are commonly associated with the toe tips (ROI 3 and 4), while in alpacas, the lateral edges of the foot show the greatest pressures (ROI 2 and 5). Thus, our results highlight that fat pads may also play a mechanical role in in reducing pressures under the foot.

Many morphological studies describing the camels foot, attribute its function towards a role in preventing the feet from sinking into soft granular sand e.g.^36,44,45^, often based on anecdotes rather than empirical measures. Yet the few studies which have detailed camel distribution have noted that camels do not appear to avoid rocky, range habitats as previously suggested^56^. Other studies have suggested, based on fossil evidence of *Paracamelus*, that the dromedary camel may have descended from a larger boreal browser, whose fat pads evolved as an adaptation in cold climates to traverse over snow^37^—much like humans wearing snow shoes^57,58^. While having larger fat pads would certainly reduce the tendency for feet to sink into sand or snow^59^, the relative influences of body mass and habitat on fat pad function are difficult to unravel.

**Figure 6:**
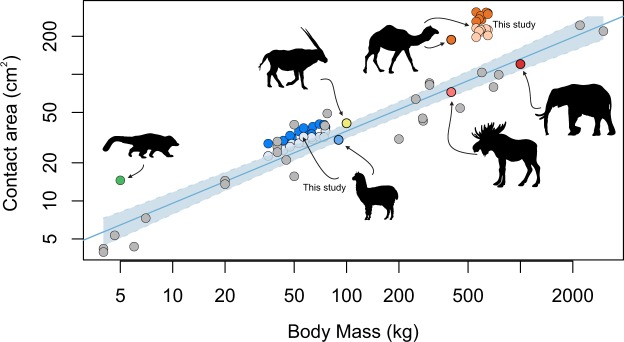
Contact area and body mass for 33 species of mammals based on Michilsens *et al*.^30^ and pers. comm. Michilsens. Linear regression and 95% confidence intervals are shown in the shaded region. Species of interest are indicated with silhouettes. Data from the current study is labelled, with dark colours representing forefeet and light colours representing hindfeet.

The link between fat pad size and habitat may be more evident when exploring the relative size of the pad with respect to body mass. A study by Michilsens, *et al*.^30^, which adopted similar experimental methods to those described here, shows camels do indeed have higher residual pad areas in comparison to other species, supporting the ‘soft substrate’ hypothesis (Fig. 6). Further, the distribution for wild camels is across much of Northern Africa and East Asia, which contains large areas of sandy deserts^60^, and following the introduction of camels to Australia approximately 100 years ago, the two areas of the highest density are now centred in the Simpson Desert, and the Great Sandy Desert - suggesting a pre-adaptation to these desert environments^56^. Yet other species, which share similar distributions to the wild populations of camels, show a distinct lack of enlarged fat pads in response to the soft granular media over which they walk. For example, the Scimitar horned oryx (*Oryx dammah*), shares a similar distribution across Northern Africa, but its foot size does not appear exaggerated for its body size, as seen in camels. Similarly the moose from Northern Canada, which has a near identical body mass to camels, has a foot area less than half that of the latter, despite living in an area frequently covered by snow, hypothesised to be the driving factor behind the enlarged fat pad in camels^37^. Finally, among the sample by Michilsens, *et al*.^30^, the species with the second largest pad area (relative to body mass) is the South American Coati, a semi-arboreal member of the racoon family, which lives in the lowland forests east of the Andes, not an area known for sandy soil. Thus, there appears to be weak or mixed evidence for the role of fat pads in habitat specialization. Alternative hypotheses have been proposed, intriguingly by tire manufacturers, who suggested that the broad fat pad of camels act to prevent sinking into soft sand via an increase in pressure along the outer edge of the pad and reduced pressure in the centre, which may act to prevent sand from moving out from underneath the foot^61^. However, this does not appear to be supported by our pressure data in camels which shows similar pressures at the centre of the pad as compared to the lateral and posterior edges.

Other hypotheses concerning the large pad of the camel have been associated with its unusual gait. Unlike most quadrupeds, which use a trotting gait where diagonal limb pairs are used in conjunction, camels appear to prefer a pacing gait where ipsilateral limbs contact the ground in unison^19,62,63^. This gait causes greater lateral instability, for example, riders avoid using horses which have been trained to use this gait^62^, and it was proposed that the large pads of camels, along with increased abductor muscle size would help to counteract this side-to-side movement^62^. Yet if this is the case for camels, we might expect higher pressures on the lateral edges of the foot in comparison to central or medial regions. Yet, here we find that these pressure patterns to be absent in the camel, yet are curiously present in the alpaca, which unlike the camel, uses the more common trotting gait, or “pace-like walk”^64–66^.

Thus, the presence of fat pads in camels appears to be linked to multiple functional roles. Here, we provide strong evidence for fat pads’ role in the reduction of relative peak locomotor pressures, reduction in localised pressures underneath the foot, and reductions in the loading rates associated with locomotion. These stress and pressure reduction strategies likely become even more important as animals increase in size and previous evidence suggests a strong positive association between pad area and body mass^30,67^. Further research into the relationship between animal speed and fat pad form-function will provide insight into the evolution of stress reduction strategies among mammals. Here we also highlight that the association between fat pads and habitat remains unclear. Often referred to as a key adaptation to desert environments, the use of fat pads for camels to transverse soft granular media, appears to lack strong comparative support, and may instead have been exploited as an exaptation, which along with a suite of other physiological characteristics of camels, allow them to survive in desert environments.

## Methods

### Subjects

Eight adult domestic dromedary camels (*Camelus dromedarius*) from natural bushland in the James Ranges, Alice Springs, Australia (Camels Australia, Stuart Well, Alice Springs) and twelve Suri alpacas (*Vicugna paccos*) from a private farm with natural grasslands in Dayboro, Queensland, Australia were included in the study. The animals ranged in body mass from 584–685 kg (camels; Supplementary Table 1) and 34–74 kg (alpacas; Supplementary Table 2). All animals were healthy with no known foot pathologies, trimmed their feet naturally and did not receive foot care from the keepers. Camels participated in short educational tourist rides around the enclosure. Animal keepers provided written consent and all experiments were approved by The University of Queensland Animal Ethics Committee (SBS/309/13/REG). An additional permit was issued to Dr Panagiotopoulou to conduct research within the Northern Territory in accordance to section 47 Animal Welfare Act.

### Data collection

Experiments were conducted at the farms and the experimental protocol is similar to that described by^14–16^. Foot pressure data was collected using two custom designed pressure platforms (Zebris Medical GmbH, Biomechanix, Munich). Sampling frequency of each platform was 100 Hz; the outer dimensions of each platform were 0.605 m (width) and 2.122 m (length) with a 48 (width) by 160 (length) array of sensors to total 7680 for each platform^15^. Each individual sensor was 1.27 cm by 1.27 cm. The pressure platforms were placed on level ground and aligned parallel to each other with their long axis in the direction of travel (Fig. 1a). For both camels and alpacas, a 0.5 cm thick rubber mat was placed on top of the plates to protect them from damage and to avoid recognition of the plates by the animals. For the camel experiments, we also spread a small amount of sand on the rubber mat to minimise sun reflections, which intimidated the camels and prevented them from moving over the experimental set-up. The plates were calibrated prior to commencing each data collection session.

All animals were trained to walk over the set-up by their keepers via a lead approximately 5 times each. To enable speed measurements, we placed two black markers on the hip and shoulder joints. Speed was measured using a GOPRO Hero 3 camera (GoPro Inc., San Mateo, California, USA) at 30 frames per second placed 2 m away, perpendicular to the walkway. Unsteady or unstable trials, such as when the animals stopped mid-way, or decelerated/accelerated, were excluded from further analysis.

### Data processing

Raw foot pressure data (*x*, *y*, time) were exported from the Zebris system and processed in Canopy v. 1.4.1 (Enthought Inc., Austin, TX, USA) using SciPy v. 0.14, NumPy 1.8.1 and Matplotlib 1.4. Each time step was represented by a 96 by 160 matrix of pressure pixels, corresponding to each sensor in the array. At each time step, groups of non-zero pressure voxels were manually classified as fore left (FL), fore right (FR), hind left (HL), hind right (HR) foot falls. From the (time averaged) mean foot pressure voxel arrays, we selected seven anatomical regions of interest (ROIs) (Fig. 1b). ROIs 1–3 and ROIs 4–6 represented the medial and lateral part of the foot (Fig. 1b), respectively. ROIs 3 & 4 represented the cranial (anterior) portion at the foot with ROI 7 located in the centre of the foot. The pressures (kPa) for each trial and foot were determined for a 1-pixel area for each digitized ROI^15^.

To determine the time-varying vertical ground reaction forces, we multiplied the pressure for each pixel by its area (1.27 cm * 1.27 cm) to extract the localised force and then summed these forces under each foot for each time point during the stance phase. Time-varying area was determined as the number of non-zero pixels at each time point multiplied by the area of a single pixel. Time-varying pressure was determined by dividing the force by the area at each time step.

### Statistics

Data for mass, velocity, contact area and pressure were linearized using log_10_-transformation. For all analyses a within-subject design was used, including subject as a random factor. The effects of mass, velocity, and species on peak vertical ground reaction force, contact area, and stress were determined using a linear mixed effects model using the lme.R function from the nlme package (Pinheiro *et al*., 2017) in R (v3.5.2, *Eggshell Igloo*, Vienna, Austria). To examine variation between factors we specified the model with lme.R function, and then used glht.R function from the multcomp package^68^ to perform Tukey post hoc tests, correcting the P-values using the Bonferroni adjustment method. Raw pressure data and the code for all analyses are accessible via fig share. (https://figshare.com/articles/Clemente_et_al_2019_Camel_foot_morphology/8293253).

## Supplementary information

Supplementary material.

## Data Availability

Raw vGRF, contact areas and video data and the code for all analyses are accessible via fig share. (https://figshare.com/articles/Clemente_et_al_2019_Camel_foot_morphology/8293253).
