# Supplementary material for: Biomechanical insights into the role of foot pads during locomotion in camelid species
Source: Sci Rep. 2020 Mar 2;10:3856. doi: 10.1038/s41598-020-60795-9 (PMC7051995; doi:10.1038/s41598-020-60795-9)
Supplement: Supplementary file 1 — Supplementary material. [file 41598_2020_60795_MOESM1_ESM.pdf]

Biomechanical insights into the role of foot pads during locomotion in camelid species

Christofer J. Clemente[1,2] , Taylor J.M. Dick[2], Christopher L. Glen[2], Olga Panagiotopoulou[2,3]

1. School of Science and Engineering, University of the Sunshine Coast, Sippy Downs, Australia.

2. School of Biomedical Sciences, University of Queensland, St Lucia, Australia

3. Monash Biomedicine Discovery Institute, Department of Anatomy and Developmental Biology, Monash University, Melbourne, Australia

Corresponding authors:
Dr Christofer Clemente (cclement@usc.edu.au)
Dr Olga Panagiotopoulou (olga.panagiotopoulou@monash.edu)

Table of contents

Supplementary Material Hindlimb Analysis 3
Supplementary Figure 1. Pressure distribution patterns for Camels. 5
Supplementary Figure 2. Pressure distribution patterns for Alpacas. 6
Supplementary Table 1. Subject characteristics dromedary camels (Camelus dromedarius). 7
Supplementary Table 2. Subject characteristics Suri alpacas (Vicugna paccos). 8
Supplementary Table 3 – Statistical results 9

__Supplementary Material Hindlimb Analysis__

Vertical Ground Reaction Forces during movement

When including mass, velocity, and species in a linear mixed effects model, peak vertical ground reaction forces in the hindlimb were significantly affected by mass ($F_{1,16}$ = 4,667, *p*<0.001) and velocity ($F_{1,914}$ = 352, *p*<0.001), but not species ($F_{1,16}$ = 0.1, *p*=0.758). Further, there was a significant interaction between mass and velocity ($F_{1,914}$= 76, *p*<0.001) and a significant interaction between mass, velocity, and species ($F_{1,914}$= 4.18, *p*=0.041). To explore this further, we looked at the effects of mass and velocity on hindlimb peak vertical ground reaction force in each species independently. Among camels, peak vertical ground reaction force increased with $M^{1.34}$ (CIS: 0.44-2.18) whilst in alpacas, peak vertical ground reaction force increased with $M^{0.85}$ (CIS: 0.61-1.09). In camels, there was no effect of velocity on peak vertical ground reaction force (slope velocity: 0.02; CIS: -0.05-0.09) whilst in alpaca's, peak vertical ground reaction force increased significantly with increases in velocity (slope velocity: 0.43; CIS: 0.39-0.48). When we normalized peak vertical ground reaction force for body mass, there was no significant effect of velocity on mass-corrected peak vertical ground reaction force in camels (velocity slope: 0.01; CIS: -0.06-0.08) while for alpacas we found that mass-corrected peak verticalground reaction force increased with velocity (velocity slope: 0.43; CIS: 0.39-0.48).

Contact Area during movement

Exploring contact areas while including mass, velocity, and species in a linear-mixed effects model revealed a significant effect of mass ($F_{1,16}$ = 9,702, *p*<0.001), velocity ($F_{1,\,914}$= 136, *p*<0.001), and species ($F_{1,16}$= 32, *p*<0.001), with a significant interaction between velocity and mass ($F_{1,914}$ = 31, *p*<0.001), but no other interaction was significant. To understand this further, we analyzed the effects of mass on foot contact area in camels and alpacas separately. Camels and alpacas show a similar increase in foot contact area with body mass. Among camels, contact area increases with $M^{0.06}$ (CIS: -0.67-0.78), but this effect was not significant (*p*=0.85), whereas among alpacas, contact area increased significantly with $M^{0.49}$ (CIS: 0.37-0.59; $F_{1,10}$= 113, *p*<0.001). However, the intercept of the relationship between contact area and mass was greater in camels than alpacas, indicating that at any given body mass, foot contact area is greater in camels than alpacas. Further comparing contact area with velocity among camels using linear mixed effects models, revealed no change in contact area with velocity (velocity slope: 0.0, CIS: -0.04-0.04, $F_{1,294}$= 0, *p*=0.972). Similarly, in alpacas, there was a significant increase in contact area with velocity (velocity slope: 0.09, CIS: 0.02-0.16, $F_{1,622}$= 6.6, *p*=0.010).

Pressures during movement

Combining these data to understand pressure variation with mass, velocity, and species revealed a significant effect of mass ($F_{1,16}$ = 73, *p*<0.001), velocity ($F_{1,914}$ = 54, *p*<0.001), and species ($F_{1,16}$ = 16, *p*=0.001), with a significant interaction of mass and velocity ($F_{1,914}$ = 6.8, *p*=0.009).This significant effect of species suggests that camels have an absolutely lower pressure at any given mass or velocity, when compared to alpacas. When we examine camels and alpacas independently, among camels there was a significant relationship between mass and pressure ($M^{0.88}$, CIS: 0.09-1.67, $F_{1,6}$ = 7.4, *p*=0.034), whereas in alpacas, pressure significantly increased with mass ($M^{0.27}$, CIS: 0.14-0.45, , $F_{1,10}$ = 19, *p*=0.001). Comparing pressure and velocity independently revealed a decrease in pressure with velocity in camels (velocity slope: 0.06, CIS: -0.08-0.20), *p*=0.409) whereas in alpacas, there was a significant increase in pressure with velocity (velocity slope: 0.19, CIS: 0.15-0.23, $F_{1,622}$= 89, *p*<0.001).

Stance Durations

Exploring forelimb stance duration while including mass, velocity, and species in a linear-mixed effects model revealed a significant effect of mass ($F_{1,16}$ = 495, *p*<0.001), velocity ($F_{1,1684}$ = 1235, *p*<0.001), and species ($F_{1,16}$ = 7.4, *p*=0.015) and a significant interaction of both mass and velocity ($F_{1,1684}$ = 57, *p*<0.001) and mass, velocity, and species ($F_{1,1684}$ = 176, *p*<0.001). The significant effect of species suggests that camels have an absolutely greater stance time at any given mass or velocity, when compared to alpacas.

Within camels (*p*=0.864) and alpacas (*p*=0.413) independently, there was no significant effect of mass on stance duration. However, for camels there was a significant decrease in stance duration with velocity (velocity slope: -0.27; CIs: -0.40-(-0.14), $F_{1,389}$= 16, *p*<0.001). Alpacas also showed a decrease, in stance duration with velocity, although to a significantly greater extent than camels (velocity slope: -0.65, CIS: -0.69-(-0.62), $F_{1,1297}$= 1579, *p*<0.001).

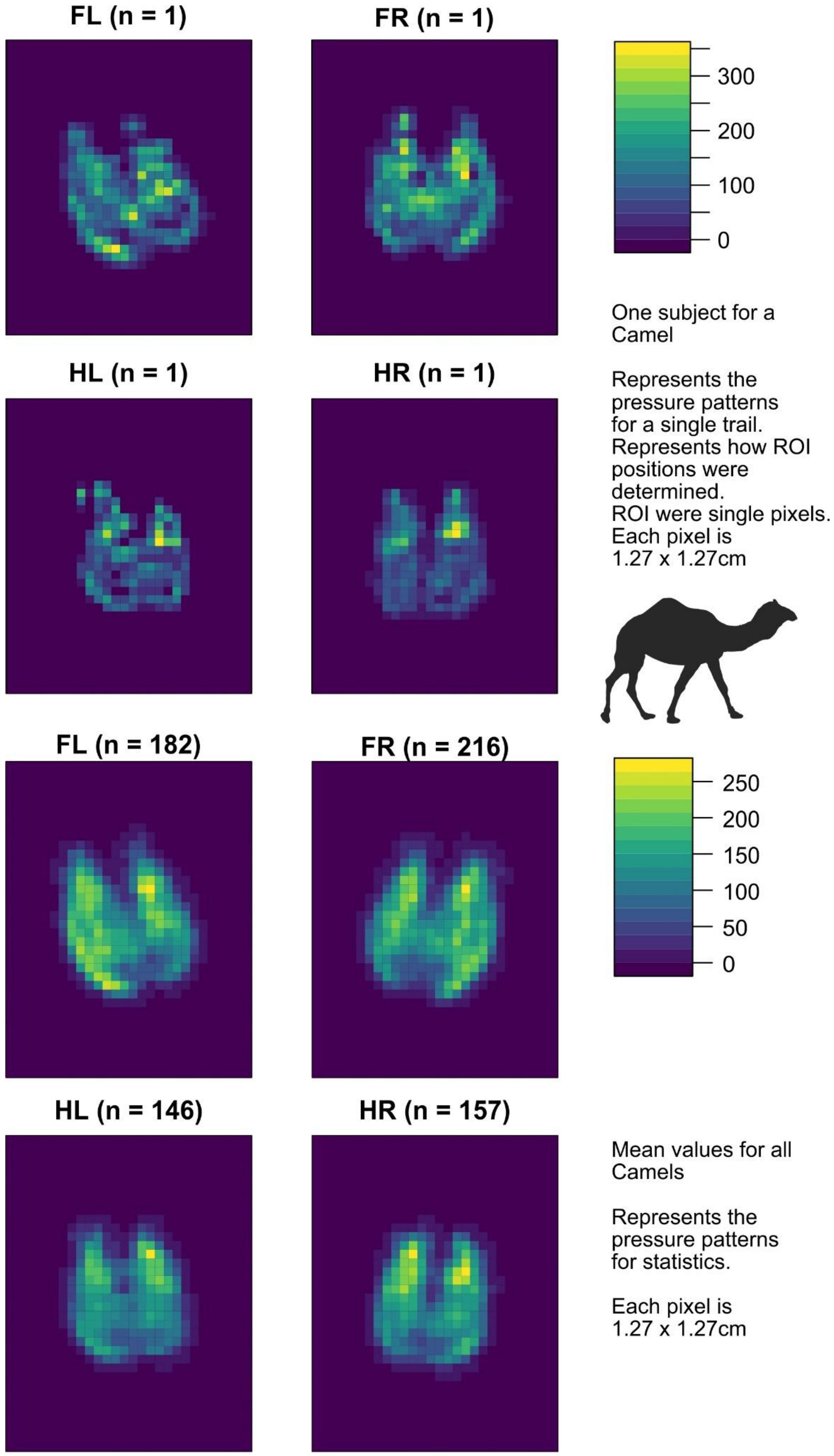

Supplementary Figure 1. Pressure distribution patterns for Camels.

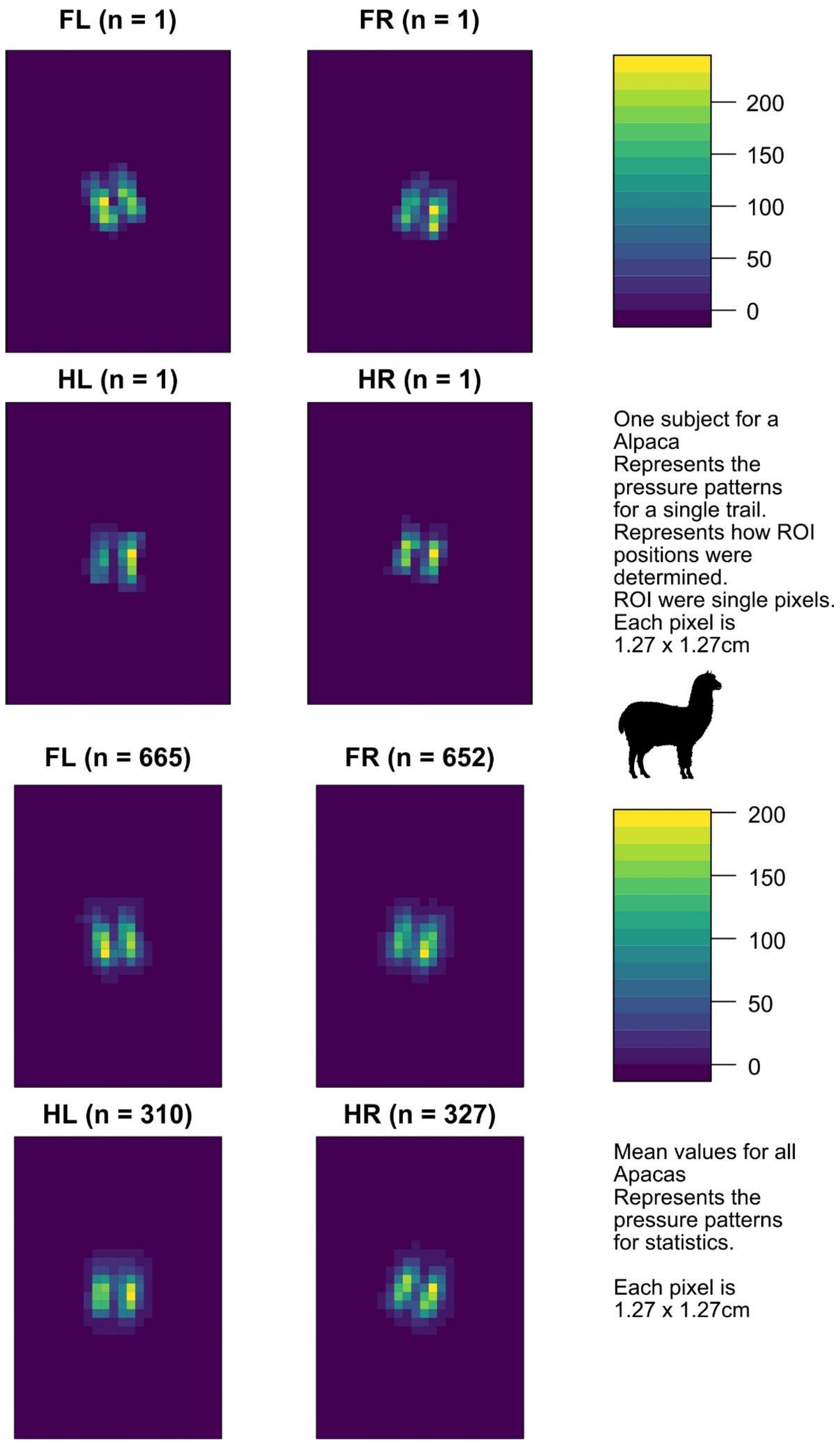

Supplementary Figure 2. Pressure distribution patterns for Alpacas.

Table 1. Subject characteristics dromedary camels (*Camelus dromedarius*).

**Subject ID**	**0 Banshee**	**1 Bull**	**2 Burt**	**3 Crissy**	**4 Effie**	**5 Flossy**	**6 Mar**	**7 Sparky**
Sex	Female	Male	Male	Female	Female	Female	Male	Male
Body mass (kg)	684	584	668	628	620	606	584	684
Hip height (m)	1.55	1.70	1.60	1.60	1.52	1.57	1.70	1.75
Mean Froude number	0.13	0.14	0.17	0.12	0.14	0.09	0.12	0.11
Mean velocity (ms^{-1})	1.42	1.50	1.60	1.35	1.42	1.21	1.41	1.36
Mean maximum pressure (KPa) Fore Left 1	148	125	141	147	135	157	144	169
Mean maximum pressure (KPa) Fore Right 2	110	95	103	100	98	98	108	118
Mean maximum pressure (KPa) Hind Left 3	135	136	142	151	138	158	134	158
Mean maximum pressure (KPa) Hind Right 4	113	85	101	97	102	110	196	120
Number of steps	108	96	99	89	116	86	67	40
Trials (multiple steps)	56	68	68	53	51	47	52	46
Steps, Fore Left	25	26	19	22	29	27	24	10
Steps, Fore Right	30	25	31	29	36	31	16	18
Steps, Hind Left	27	25	21	15	21	13	18	6
Steps, Hind Right	26	20	28	23	30	15	9	6

Table 2. Subject characteristics Suri alpacas (*Vicugna paccos*).

**Subject ID**	**0 April**	**1 Ceasar**	**2 George**	**3 Giselle**	**4 Goldie**	**5 Hurricane**	**6 Khamsin**	**7 Nureyev**	**8 Opal**	**9 Prada**	**10 Smoky**	**11 Sophie**
Sex	Female	Male	Male	Female	Female	Male	Male	Female	Female	Male	Male	Female
Body mass (kg)	57	43	35	48	64	70	47	63	53	56	76	74
Hip height (m)	0.79	0.83	0.75	0.75	0.85	0.93	0.82	0.87	0.85	0.87	0.91	0.86
Mean Froude number	0.19	0.18	0.22	0.21	0.15	0.13	0.17	0.14	0.20	0.20	0.14	0.18
Mean velocity (ms^{-1})	1.20	1.21	1.19	1.22	1.11	1.09	1.15	1.07	1.25	1.28	1.09	1.23
Mean maximum pressure (KPa) Fore Left 1	116	101	92	107	114	126	100	134	114	133	135	137
Mean maximum pressure (KPa) Fore Right 2	116	104	94	99	113	131	102	129	106	132	130	138
Mean maximum pressure (KPa) Hind Left 3	92	85	74	87	89	87	74	86	81	93	93	94
Mean maximum pressure (KPa) Hind Right 4	90	83	70	83	88	86	72	84	80	94	91	95
Number of steps	195	141	121	147	212	186	184	198	98	78	203	191
Trials (multiple steps)	40	39	36	40	40	40	40	40	40	45	40	40
Steps, Fore Left	75	46	46	51	75	67	59	55	30	25	69	67
Steps, Fore Right	71	55	46	51	75	64	48	56	33	23	67	63
Steps, Hind Left	19	21	13	23	29	26	34	42	19	19	33	32
Steps, Hind Right	30	19	16	22	33	29	43	45	16	11	34	29

Supplementary Table 3 – Statistical results

**Camel leg (fore, hind) and foot (left, right) comparison**	<pre>m2 = lme(log10(GRF) ~ Leg+Foot, data=camel, random = ~1|as.factor(Subject2),<br>method ='ML', na.action=na.exclude)<br><br>numDF denDF F-value p-value<br>(Intercept) 1 691 68026.63 <.0001<br>Leg 1 691 15827.47 <.0001<br>Foot 1 691 0.46 0.4988</pre>
**Alpaca leg (fore, hind) and foot (left, right) comparison**	<pre>m2 = lme(log10(GRF) ~ Leg+Foot, data=alpaca, random = ~1|as.factor(Subject2),<br>method ='ML', na.action=na.exclude)<br><br>numDF denDF F-value p-value<br>(Intercept) 1 1940 8554.606 <.0001<br>Leg 1 1940 9266.571 <.0001<br>Foot 1 1940 0.083 0.7736</pre>

Vertical Ground Reaction Forces during movement

<table>
<tr>
<td>Fore limbs</td>
<td>
<pre>
m2 = lme(log(GRF) ~ log(Mass)*log(Velocity)*Species, data=forelimb2, random =
~1|as.factor(Subject2), method ='ML', na.action=na.exclude)

                                numDF denDF F-value p-value
(Intercept)   1 1684 418367.7 <.0001
log(Mass)   1   16 11946.4 <.0001
log(Velocity)   1 1684   16.0 0.0001
Species   1   16   0.1 0.7313
log(Mass):log(Velocity)   1 1684   0.1 0.7927
log(Mass):Species   1   16   0.0 0.9774
log(Velocity):Species   1 1684   7.6 0.0059
log(Mass):log(Velocity):Species   1 1684   1.8 0.1812
</pre>
</td>
</tr>
<tr>
<td>Hind limbs</td>
<td>
<pre>
m2 = lme(log(GRF) ~ log(Mass)*log(Velocity)*Species, data=hindlimb2, random =
~1|as.factor(Subject2), method ='ML', na.action=na.exclude)

                               numDF denDF   F-value p-value
(Intercept)   1   914 167923.65 <.0001
log(Mass)   1   16   4643.29 <.0001
log(Velocity)   1   914   352.57 <.0001
Species   1   16   0.10 0.7512
log(Mass):log(Velocity)   1   914   76.12 <.0001
log(Mass):Species   1   16   1.49 0.2397
log(Velocity):Species   1   914   0.13 0.7217
log(Mass):log(Velocity):Species   1   914   4.18 0.0413
</pre>
</td>
</tr>
</table>

Camels only.

Vertical Ground Reaction Forces with *Mass* – Camels

**Fore limbs**	`m2 = lme(log10(GRF) ~ log10(Mass), data=forelimb2[which(forelimb2$Species=='Camel'),], random = ~1|as.factor(Subject2), method ='ML', na.action=na.exclude)`<br><br>`anova(m2)`<br>`numDF denDF F-value p-value`<br>`(Intercept) 1 390 237342.1 <.0001`<br>`log10(Mass) 1 6 12.6 0.0121`
**Hind limbs**	`m3 = lme(log10(GRF) ~ log10(Mass), data=hindlimb2[which(hindlimb2$Species=='Camel'),], random = ~1|as.factor(Subject2), method ='ML', na.action=na.exclude)`<br><br>`anova(m3)`<br>`numDF denDF F-value p-value`<br>`(Intercept) 1 295 120603.84 <.0001`<br>`log10(Mass) 1 6 13.53 0.0104`

Vertical Ground Reaction Forces with *Velocity* – Camels

**Fore limbs**	`m2 = lme(log10(GRF) ~ log10(Velocity), data=forelimb2[which(forelimb2$Species=='Camel'),], random = ~1|as.factor(Subject2), method ='ML', na.action=na.exclude)`<br><br>`anova(m2)`<br>`numDF denDF F-value p-value`<br>`(Intercept) 1 389 90752.79 <.0001`<br>`log10(Velocity) 1 389 0.48 0.4867`

**Hind limbs**	`m3 = lme(log10(GRF) ~ log10(Velocity), data=hindlimb2[which(hindlimb2$Species=='Camel'),], random = ~1|as.factor(Subject2), method ='ML', na.action=na.exclude)`<br><br>`anova(m3)`<br>`numDF denDF F-value p-value`<br>`(Intercept) 1 294 44069.45 <.0001`<br>`log10(Velocity) 1 294 0.20 0.657`

Alpacas Only

Vertical Ground Reaction Forces with *Mass* – Alpacas

**Fore limbs**	`m4 = lme(log10(GRF) ~ log10(Mass), data=forelimb2[which(forelimb2$Species=='Alpaca'),], random = ~1|as.factor(Subject2), method ='ML', na.action=na.exclude)`<br><br>`anova(m4)`<br>`numDF denDF F-value p-value`<br>`(Intercept) 1 1305 186307.44 <.0001`<br>`log10(Mass) 1 10 256.21 <.0001`
**Hind limbs**	`m5 = lme(log10(GRF) ~ log10(Mass), data=hindlimb2[which(hindlimb2$Species=='Alpaca'),], random = ~1|as.factor(Subject2), method ='ML', na.action=na.exclude)`<br><br>`anova(m5)`<br>`numDF denDF F-value p-value`<br>`(Intercept) 1 625 51817.31 <.0001`<br>`log10(Mass) 1 10 63.08 <.0001`

Vertical Ground Reaction Forces with *Velocity* – Alpacas

**Fore limbs**	`m4 = lme(log(GRF) ~ log10(Velocity), data=forelimb2[which(forelimb2$Species=='Alpaca'),], random = ~1|as.factor(Subject2), method ='ML', na.action=na.exclude)`<br><br>`anova(m4)`<br>`                numDF denDF  F-value p-value`<br>`(Intercept)         1  1297 8270.304  <.0001`<br>`log10(Velocity)     1  1297   15.555   1e-04`
**Hind limbs**	`m5 = lme(log(GRF) ~ log10(Velocity), data=hindlimb2[which(hindlimb2$Species=='Alpaca'),], random = ~1|as.factor(Subject2), method ='ML', na.action=na.exclude)`<br><br>`anova(m5)`<br>`                numDF denDF  F-value p-value`<br>`(Intercept)         1   622 8571.181  <.0001`<br>`log10(Velocity)     1   622  356.290  <.0001`

<u>**Foot Contact Area during movement**</u>

**Fore limbs**	<pre>m2 = lme(log10(Cont) ~ log10(Mass)*log10(Velocity)*Species, data=forelimb2,<br>random = ~1|as.factor(Subject2), method ='ML', na.action=na.exclude)<br><br>anova(m2)<br>numDF denDF F-value p-value<br>(Intercept) 1 1684 188850.22 <.0001<br>log10(Mass) 1 16 9558.87 <.0001<br>log10(Velocity) 1 1684 0.68 0.4091<br>Species 1 16 47.02 <.0001<br>log10(Mass):log10(Velocity) 1 1684 0.35 0.5526<br>log10(Mass):Species 1 16 0.03 0.8718<br>log10(Velocity):Species 1 1684 13.32 0.0003<br>log10(Mass):log10(Velocity):Species 1 1684 0.68 0.4086</pre>
**Hind limbs**	<pre>m2 = lme(log10(Cont) ~ log10(Mass)*log10(Velocity)*Species, data=hindlimb2,<br>random = ~1|as.factor(Subject2), method ='ML', na.action=na.exclude)<br><br>anova(m2)<br>numDF denDF F-value p-value<br>(Intercept) 1 914 191925.36 <.0001<br>log10(Mass) 1 16 9702.79 <.0001<br>log10(Velocity) 1 914 136.65 <.0001<br>Species 1 16 32.09 <.0001<br>log10(Mass):log10(Velocity) 1 914 30.86 <.0001<br>log10(Mass):Species 1 16 2.29 0.1496<br>log10(Velocity):Species 1 914 1.11 0.2921<br>log10(Mass):log10(Velocity):Species 1 914 0.05 0.8164</pre>

foot contact area with mass – camels

**Fore limbs**	`m2 = lme(log10(Cont) ~ log10(Mass), data=forelimb2[which(forelimb2$Species=='Camel'),], random = ~1|as.factor(Subject2), method ='ML', na.action=na.exclude)`<br><br>`anova(m2)`<br>`numDF denDF F-value p-value`<br>`(Intercept) 1 390 93788.30 <.0001`<br>`log10(Mass) 1 6 2.71 0.1511`
**Hind limbs**	`m3 = lme(log10(Cont) ~ log10(Mass), data=hindlimb2[which(hindlimb2$Species=='Camel'),], random = ~1|as.factor(Subject2), method ='ML', na.action=na.exclude)`<br><br>`anova(m3)`<br>`numDF denDF F-value p-value`<br>`(Intercept) 1 295 85773.60 <.0001`<br>`log10(Mass) 1 6 0.04 0.852`

foot contact area with velocity – camels

**Fore limbs**	`m2 = lme(log10(Cont) ~ log10(Velocity), data=forelimb2[which(forelimb2$Species=='Camel'),], random = ~1|as.factor(Subject2), method ='ML', na.action=na.exclude)`<br><br>`anova(m2)`<br>`numDF denDF F-value p-value`<br>`(Intercept) 1 389 68878.16 <.0001`<br>`log10(Velocity) 1 389 0.52 0.4713`

**Hind limbs**	`m3 = lme(log10(Cont) ~ log10(Velocity), data=hindlimb2[which(hindlimb2$Species=='Camel'),], random = ~1|as.factor(Subject2), method ='ML', na.action=na.exclude)`<br><br>`anova(m3)`<br>`                numDF denDF  F-value p-value`<br>`(Intercept)         1   294 85339.43  <.0001`<br>`log10(Velocity)     1   294     0.00  0.9724`

foot contact area with mass – alpacas

**Fore limbs**	`m2 = lme(log10(Cont) ~ log10(Mass), data=forelimb2[which(forelimb2$Species=='Alpaca'),], random = ~1|as.factor(Subject2), method ='ML', na.action=na.exclude)`<br><br>`anova(m2)`<br>`            numDF denDF  F-value p-value`<br>`(Intercept)     1  1305 92978.15  <.0001`<br>`log10(Mass)     1    10    68.76  <.0001`
**Hind limbs**	`m3 = lme(log10(Cont) ~ log10(Mass), data=hindlimb2[which(hindlimb2$Species=='Alpaca'),], random = ~1|as.factor(Subject2), method ='ML', na.action=na.exclude)`<br><br>`anova(m3)`<br>`            numDF denDF   F-value p-value`<br>`(Intercept)     1   625 113968.97  <.0001`<br>`log10(Mass)     1    10    113.39  <.0001`

foot contact area with velocity – alpacas

**Fore limbs**	`m2 = lme(log10(Cont) ~ log10(Velocity), data=forelimb2[which(forelimb2$Species=='Alpaca'),], random = ~1|as.factor(Subject2), method ='ML', na.action=na.exclude)`<br><br>`anova(m2)`<br>`                 numDF denDF   F-value p-value`<br>`(Intercept)          1  1297 13592.372  <.0001`<br>`log10(Velocity)      1  1297     0.498  0.4805`
**Hind limbs**	`m3 = lme(log10(Cont) ~ log10(Velocity), data=hindlimb2[which(hindlimb2$Species=='Alpaca'),], random = ~1|as.factor(Subject2), method ='ML', na.action=na.exclude)`<br><br>`anova(m3)`<br>`                 numDF denDF   F-value p-value`<br>`(Intercept)          1   622 10403.689  <.0001`<br>`log10(Velocity)      1   622   119.325  <.0001`

Pressures during movement

**Fore limbs**	<pre>m2 = lme(log10(Stress) ~ log10(Mass)*log10(Velocity)*Species, data=forelimb2,<br>random = ~1|as.factor(Subject2), method ='ML', na.action=na.exclude)<br><br>anova(m2)<br>numDF denDF F-value p-value<br>(Intercept) 1 1684 37393.48 <.0001<br>log10(Mass) 1 16 84.72 <.0001<br>log10(Velocity) 1 1684 0.00 0.9670<br>Species 1 16 31.59 <.0001<br>log10(Mass):log10(Velocity) 1 1684 0.02 0.8888<br>log10(Mass):Species 1 16 0.01 0.9138<br>log10(Velocity):Species 1 1684 2.16 0.1417<br>log10(Mass):log10(Velocity):Species 1 1684 0.01 0.9051</pre>
**Hind limbs**	<pre>m3 = lme(log10(Stress) ~ log10(Mass)*log10(Velocity)*Species, data=hindlimb2,<br>random = ~1|as.factor(Subject2), method ='ML', na.action=na.exclude)<br><br>anova(m3)<br>numDF denDF F-value p-value<br>(Intercept) 1 914 36976.53 <.0001<br>log10(Mass) 1 16 73.55 <.0001<br>log10(Velocity) 1 914 54.04 <.0001<br>Species 1 16 16.02 0.0010<br>log10(Mass):log10(Velocity) 1 914 6.80 0.0092<br>log10(Mass):Species 1 16 3.81 0.0687<br>log10(Velocity):Species 1 914 0.79 0.3730<br>log10(Mass):log10(Velocity):Species 1 914 1.40 0.2377</pre>

pressures with mass – camels

**Fore limbs**	`m2 = lme(log10(Stress) ~ log10(Mass), data=forelimb2[which(forelimb2$Species=='Camel'),], random = ~1|as.factor(Subject2), method ='ML', na.action=na.exclude)`<br><br>`anova(m2)`<br>`             numDF denDF   F-value p-value`<br>`(Intercept)      1   390 13738.225  <.0001`<br>`log10(Mass)      1     6     1.616  0.2508`
**Hind limbs**	`m3 = lme(log10(Stress) ~ log10(Mass), data=hindlimb2[which(hindlimb2$Species=='Camel'),], random = ~1|as.factor(Subject2), method ='ML', na.action=na.exclude)`<br><br>`anova(m3)`<br>`             numDF denDF   F-value p-value`<br>`(Intercept)      1   295 14066.435  <.0001`<br>`log10(Mass)      1     6     7.453  0.0342`

pressures with velocity – camels

**Fore limbs**	`m2 = lme(log10(Stress) ~ log10(Velocity), data=forelimb2[which(forelimb2$Species=='Camel'),], random = ~1|as.factor(Subject2), method ='ML', na.action=na.exclude)`<br><br>`anova(m2)`<br>`                 numDF denDF   F-value p-value`<br>`(Intercept)          1   389 11249.611  <.0001`<br>`log10(Velocity)      1   389     0.117  0.7321`

**Hind limbs**	<pre>m3 = lme(log10(Stress) ~ log10(Velocity),<br>data=hindlimb2[which(hindlimb2$Species=='Camel'),], random =<br>~1|as.factor(Subject2), method ='ML', na.action=na.exclude)<br><br>anova(m3)<br>numDF denDF F-value p-value<br>(Intercept) 1 294 6850.946 <.0001<br>log10(Velocity) 1 294 0.681 0.4099</pre>

pressures with mass – alpacas

**Fore limbs**	<pre>m2 = lme(log10(Stress) ~ log10(Mass),<br>data=forelimb2[which(forelimb2$Species=='Alpaca'),], random =<br>~1|as.factor(Subject2), method ='ML', na.action=na.exclude)<br><br>anova(m2)<br>numDF denDF F-value p-value<br>(Intercept) 1 1305 24907.634 <.0001<br>log10(Mass) 1 10 54.191 <.0001</pre>
**Hind limbs**	<pre>m3 = lme(log10(Stress) ~ log10(Mass),<br>data=hindlimb2[which(hindlimb2$Species=='Alpaca'),], random =<br>~1|as.factor(Subject2), method ='ML', na.action=na.exclude)<br><br>anova(m3)<br>numDF denDF F-value p-value<br>(Intercept) 1 625 19410.947 <.0001<br>log10(Mass) 1 10 18.579 0.0015</pre>

pressures with velocity – alpacas

**Fore limbs**	<pre>m2 = lme(log10(Stress) ~ log10(Velocity),<br>data=forelimb2[which(forelimb2$Species=='Alpaca'),], random =<br>~1|as.factor(Subject2), method ='ML', na.action=na.exclude)<br><br>anova(m2)<br>numDF denDF F-value p-value<br>(Intercept) 1 1297 4477.854 <.0001<br>log10(Velocity) 1 1297 0.012 0.9128</pre>
**Hind limbs**	<pre>m3 = lme(log10(Stress) ~ log10(Velocity),<br>data=hindlimb2[which(hindlimb2$Species=='Alpaca'),], random =<br>~1|as.factor(Subject2), method ='ML', na.action=na.exclude)<br><br>anova(m3)<br>numDF denDF F-value p-value<br>(Intercept) 1 622 8314.531 <.0001<br>log10(Velocity) 1 622 89.205 <.0001</pre>

Table 4 Statistics and Tukey post hoc tests to compare among ROI

Camel fore feet.

Post hoc analyses revealed that for camels, the fore feet ROIs displayed significant variation from one another with the exception of regions ROI1 versus ROI3 and ROI7, ROI2 versus ROI4, ROI5 and ROI6, ROI3 versus ROI7, ROI4 versus ROI5, and ROI5 versus ROI6. In the camel hind feet, all pairs of ROIs varied significantly with the exception ROI1 versus ROI5, ROI2 versus ROI5 and ROI6, and ROI5 versus ROI6.

```
Camel_fore<-camel2[which(camel2$foot_lab == 'FL' | camel2$foot_lab == 'FR'),]
summary(fit<-aov(roi~roi_lab, data=Camel_fore))
```

```
              Df   Sum Sq Mean Sq F value Pr(>F)    
roi_lab        6  2783635  463939   64.59 <2e-16 ***
Residuals   2770 19896387    7183                   
---
Signif. codes:  0 '***' 0.001 '**' 0.01 '*' 0.05 '.' 0.1 ' ' 1
9 observations deleted due to missingness
```

```
TukeyHSD(fit)
```

```
Tukey multiple comparisons of means
    95% family-wise confidence level

Fit: aov(formula = roi ~ roi_lab, data = Camel_fore)

$roi_lab
             diff        lwr        upr     p adj
p2-p1  56.7685904  39.020026  74.517154 0.0000000
p3-p1  -5.0580617 -22.817771  12.701647 0.9806872
p4-p1  72.2183392  54.469775  89.966903 0.0000000
p5-p1  57.5876859  39.839122  75.336250 0.0000000
p6-p1  45.6677691  27.874328  63.461210 0.0000000
p7-p1  -7.3005051 -25.071408  10.470398 0.8897061
p3-p2 -61.8266522 -79.564008 -44.089296 0.0000000
```

```
p4-p2  15.4497487  -2.276448  33.175945 0.1351220
p5-p2   0.8190955 -16.907101  18.545292 0.9999995
p6-p2 -11.1008214 -28.871951   6.670309 0.5188341
p7-p2 -64.0690955 -81.817660 -46.320531 0.0000000
p4-p3  77.2764009  59.539045  95.013757 0.0000000
p5-p3  62.6457476  44.908392  80.383103 0.0000000
p6-p3  50.7258308  32.943570  68.508092 0.0000000
p7-p3  -2.2424433 -20.002152  15.517266 0.9997908
p5-p4 -14.6306533 -32.356850   3.095543 0.1842494
p6-p4 -26.5505701 -44.321700  -8.779440 0.0002186
p7-p4 -79.5188442 -97.267408 -61.770280 0.0000000
p6-p5 -11.9199168 -29.691047   5.851213 0.4282921
p7-p5 -64.8881910 -82.636755 -47.139627 0.0000000
p7-p6 -52.9682741 -70.761715 -35.174833 0.0000000
```

Camel hind feet

```
Camel_hind<-camel2[which(camel2$foot_lab == 'HL' | camel2$foot_lab == 'HR'),]
summary(fit<-aov(roi~roi_lab, data=Camel_hind))
```

```
              Df  Sum Sq Mean Sq F value Pr(>F)    
roi_lab        6 4616959  769493     206 <2e-16 ***
Residuals   2103 7854609    3735                   
---
Signif. codes:  0 ‘***’ 0.001 ‘**’ 0.01 ‘*’ 0.05 ‘.’ 0.1 ‘ ’ 1
11 observations deleted due to missingness
```

```
TukeyHSD(fit)
```

```
 Tukey multiple comparisons of means
    95% family-wise confidence level

Fit: aov(formula = roi ~ roi_lab, data = Camel_hind)

$roi_lab
              diff         lwr        upr     p adj
p2-p1   20.0777076   5.3636494  34.791766 0.0011447
p3-p1  119.7111921 105.0092990 134.413085 0.0000000
p4-p1   92.3239934  77.6341953 107.013791 0.0000000
p5-p1   14.3536964  -0.3361017  29.043494 0.0605131
p6-p1   20.6276238   5.9378257  35.317422 0.0007019
p7-p1  -17.7475839 -32.4985663  -2.996602 0.0071816
p3-p2   99.6334844  84.9438480 114.323121 0.0000000
p4-p2   72.2462858  57.5687543  86.923817 0.0000000
p5-p2   -5.7240113 -20.4015427   8.953520 0.9120287
p6-p2    0.5499161 -14.1276153  15.227448 0.9999998
```

p7-p2	-37.8252915	-52.5640582	-23.086525	0.0000000
p4-p3	-27.3871987	-42.0525345	-12.721863	0.0000008
p5-p3	-105.3574957	-120.0228315	-90.692160	0.0000000
p6-p3	-99.0835683	-113.7489041	-84.418232	0.0000000
p7-p3	-137.4587759	-152.1853977	-122.732154	0.0000000
p5-p4	-77.9702970	-92.6235077	-63.317086	0.0000000
p6-p4	-71.6963696	-86.3495803	-57.043159	0.0000000
p7-p4	-110.0715773	-124.7861244	-95.357030	0.0000000
p6-p5	6.2739274	-8.3792833	20.927138	0.8684715
p7-p5	-32.1012803	-46.8158274	-17.386733	0.0000000
p7-p6	-38.3752077	-53.0897548	-23.660661	0.0000000

Alpaca fore feet.

Post hoc analyses revealed that in the alpaca fore feet, all ROIs displayed significant variation from one another. In the alpaca hind feet, all pairs of ROIs varied significantly with the exception of ROI1 versus ROI6, ROI2 versus ROI5, and ROI3 versus ROI4. The medial posterior and middle (ROI1 and ROI2) and lateral posterior and middle (ROI5 and ROI6) regions of the forefeet showed moderately higher peak pressures than anterior (ROI3 and ROI4) and central (ROI7) regions.

```
alpaca_fore<-alpaca2[which(alpaca2$foot_lab == 'FL' | alpaca2$foot_lab == 'FR'),]
summary(fit<-aov(roi~roi_lab, data=alpaca_fore))
```

```
              Df   Sum Sq Mean Sq F value Pr(>F)    
roi_lab        6 17976854 2996142    1198 <2e-16 ***
Residuals   9029 22589647    2502                   
---
Signif. codes:  0 ‘***’ 0.001 ‘**’ 0.01 ‘*’ 0.05 ‘.’ 0.1 ‘ ’ 1
134 observations deleted due to missingness
```

```
TukeyHSD(fit)
```

```
  Tukey multiple comparisons of means
    95% family-wise confidence level

Fit: aov(formula = roi ~ roi_lab, data = alpaca_fore)

$roi_lab
             diff         lwr          upr     p adj
p2-p1   58.516367   52.751755   64.2809786 0.0000000
p3-p1  -57.457470  -63.269603  -51.6453376 0.0000000
p4-p1  -49.237694  -55.016727  -43.4586614 0.0000000
p5-p1   36.916367   31.151755   42.6809786 0.0000000
```

p6-p1	7.148677	1.366280	12.9310739	0.0049950
p7-p1	-63.615295	-69.451036	-57.7795541	0.0000000
p3-p2	-115.973838	-121.784879	-110.1627963	0.0000000
p4-p2	-107.754061	-113.531996	-101.9761263	0.0000000
p5-p2	-21.600000	-27.363511	-15.8364890	0.0000000
p6-p2	-51.367690	-57.148990	-45.5863904	0.0000000
p7-p2	-122.131662	-127.966316	-116.2970083	0.0000000
p4-p3	8.219776	2.394429	14.0451237	0.0006343
p5-p3	94.373838	88.562796	100.1848787	0.0000000
p6-p3	64.606148	58.777463	70.4348323	0.0000000
p7-p3	-6.157825	-12.039434	-0.2762155	0.0331607
p5-p4	86.154061	80.376126	91.9319964	0.0000000
p6-p4	56.386371	50.590692	62.1820508	0.0000000
p7-p4	-14.377601	-20.226503	-8.5286984	0.0000000
p6-p5	-29.767690	-35.548990	-23.9863904	0.0000000
p7-p5	-100.531662	-106.366316	-94.6970083	0.0000000
p7-p6	-70.763972	-76.616199	-64.9117459	0.0000000

Alpaca hind feet.

```
alpaca_hind<-alpaca2[which(alpaca2$foot_lab == 'HL' | alpaca2$foot_lab == 'HR'),]
summary(fit<-aov(roi~roi_lab, data=alpaca_hind))
```

```
              Df  Sum Sq Mean Sq F value Pr(>F)
roi_lab        6 5060069  843345   854.1 <2e-16 ***
Residuals   4244 4190732     987
---
Signif. codes:  0 ‘***’ 0.001 ‘**’ 0.01 ‘*’ 0.05 ‘.’ 0.1 ‘ ’ 1
194 observations deleted due to missingness
```

```
TukeyHSD(fit)
```

```
  Tukey multiple comparisons of means
    95% family-wise confidence level

Fit: aov(formula = roi ~ roi_lab, data = alpaca_hind)

$roi_lab
             diff        lwr         upr     p adj
p2-p1  45.6084249  40.387776  50.8290734 0.0000000
p3-p1 -35.0187351 -40.323501 -29.7139689 0.0000000
p4-p1 -35.6478960 -40.950386 -30.3454063 0.0000000
p5-p1  41.3611635  36.138471  46.5838555 0.0000000
p6-p1  -4.9658908 -10.196814   0.2650321 0.0757297
p7-p1 -47.5602533 -53.023262 -42.0972447 0.0000000
p3-p2 -80.6271601 -85.913559 -75.3407607 0.0000000
p4-p2 -81.2563210 -86.540436 -75.9722059 0.0000000
p5-p2  -4.2472615  -9.451297   0.9567741 0.1955153
p6-p2 -50.5743157 -55.786612 -45.3620198 0.0000000
```

```
p7-p2 -93.1686783 -98.613854 -87.7235026 0.0000000
p4-p3  -0.6291609  -5.996399   4.7380772 0.9998647
p5-p3  76.3798986  71.091481  81.6683161 0.0000000
p6-p3  30.0528444  24.756298  35.3493906 0.0000000
p7-p3 -12.5415182 -18.067395  -7.0156417 0.0000000
p5-p4  77.0090595  71.722925  82.2951935 0.0000000
p6-p4  30.6820053  25.387739  35.9762715 0.0000000
p7-p4 -11.9123573 -17.436048  -6.3886661 0.0000000
p6-p5 -46.3270542 -51.541397 -41.1127115 0.0000000
p7-p5 -88.9214168 -94.368552 -83.4742818 0.0000000
p7-p6 -42.5943625 -48.049390 -37.1393354 0.0000000
```
